# Acute Kidney Injury in Patients with Liver Cirrhosis: From Past to Present Definition and Diagnosis

**DOI:** 10.3390/life15081249

**Published:** 2025-08-06

**Authors:** Andreea Lungu, Georgiana-Elena Sarbu, Alexandru Sebastian Cotlet, Ilie-Andreas Savin, Ioana-Roxana Damian, Simona Juncu, Cristina Muzica, Irina Girleanu, Ana-Maria Sîngeap, Carol Stanciu, Anca Trifan, Camelia Cojocariu

**Affiliations:** 1 Department of Gastroenterology, Faculty of Medicine, “Grigore T. Popa” University of Medicine and Pharmacy, Universitatii Street No. 16, 700115 Iasi, Romania; andreea-lungu@umfiasi.ro (A.L.); georgiana-elena.sarbu@umfiasi.ro (G.-E.S.); ilie-andreas.savin@umfiasi.ro (I.-A.S.); ioana-roxana.damian@umfiasi.ro (I.-R.D.); irina.girleanu@umfiasi.ro (I.G.); ana.singeap@umfiasi.ro (A.-M.S.); carol.stanciu@umfiasi.ro (C.S.); anca.trifan@umfiasi.ro (A.T.); cojocariu.salloum@umfiasi.ro (C.C.); 2 Institute of Gastroenterology and Hepatology, “St. Spiridon” Emergency County Hospital, Bd. Independentei No. 1, 700111 Iasi, Romania; alexandru.cotlet04@gmail.com

**Keywords:** biomarkers, acute kidney disease, acute kidney injury

## Abstract

Acute kidney injury (AKI) is a serious clinical condition that is linked to markedly higher rates of morbidity and mortality in cirrhosis patients. Its diagnosis is challenging due to overlapping clinical and laboratory features among causes such as hepatorenal syndrome (HRS), acute tubular injury (ATI), and prerenal hypovolemia. In order to address the distinct pathophysiology and clinical context of cirrhosis, the definitions and classification of AKI have changed over time, moving from RIFLE and AKIN to KDIGO and ICA-AKI. Because cirrhosis patients have altered muscle mass and fluid retention, traditional markers like serum creatinine (sCr) and urine output have significant limitations. Neutrophil gelatinase-associated lipocalin (NGAL), kidney injury molecule-1 (KIM-1), interleukin-18 (IL-18), and cystatin C (CysC) are some of the new biomarkers that have shown promise in early AKI detection and in differentiating structural from functional kidney injury. NGAL and KIM-1 are sensitive indicators of tubular damage with potential prognostic implications. IL-18 reflects inflammatory injury, and CysC offers a more reliable measure of glomerular filtration. Incorporating these markers may improve early diagnosis, risk stratification, and treatment decisions, representing a key direction for future research in managing AKI in cirrhosis.

## 1. Introduction

Cirrhosis is the end stage of all chronic liver disease and is defined by the formation of regenerative nodules bounded by fibrous tissue relative to chronic liver damage. This condition is associated with portal hypertension, end-stage liver disease, and hepatocellular carcinoma (HCC) [1,2]. Cirrhosis is associated with increased mortality and morbidity because of decompensated hepatic function, which determines a huge distress in the health system [3]. This form of chronic liver disease contributes to many different types of systemic complications, including the cardiovascular, nervous, respiratory, coagulation, and renal systems [4]. About one-third of cirrhotic patients are treated as outpatients, and almost half of those admitted to the hospital with cirrhosis-related problems have AKI, which stands out due to its high incidence and its strong association with increased morbidity and mortality, making it a critical focus in the management of these patients. There is a sevenfold increase in morbidity and death in people with cirrhosis who have AKI compared to those who do not. Chronic kidney disease (CKD) is more likely to develop after recurrent AKI [5].

In most instances, hypovolemia is the predominant cause of renal hypoperfusion, which is also called prerenal AKI. Intrinsic, structural kidney damage and postrenal injury caused by urinary obstruction are the other two main causes of AKI [6,7,8]. HRS, caused by renal vasoconstriction, is a distinct cause of AKI in cirrhotic individuals because it causes renal hypoperfusion. About 30% of AKI cases in cirrhotic patients are caused by intrinsic factors (such as ATI), 15 to 20% are attributable to HRS, and less than 1% are caused by postrenal obstruction. Hypoperfusion due to hypovolemia accounts for about half of these cases. In individuals with severe liver disease, these are the most prevalent causes of AKI [7,8,9].

Given the high incidence and poor prognosis of AKI in patients with cirrhosis, this review aims to explore and clarify the current understanding of its etiology, classification, and diagnostic challenges. A central objective is to highlight the limitations of traditional diagnostic criteria in cirrhotic populations, particularly the reduced reliability of sCr and urine output due to altered physiology in these patients. In this context, the review also seeks to examine the evolution of AKI definitions—from RIFLE and AKIN to the more recent KDIGO and ICA-AKI criteria—and how they apply specifically to cirrhotic individuals.

Furthermore, this article emphasizes the emerging role of novel biomarkers such as NGAL, KIM-1, IL-18, and CysC in differentiating between functional and structural kidney injury. By synthesizing current evidence, the review intends to support more accurate diagnosis, improved risk stratification, and timely therapeutic decision-making in the management of AKI in cirrhosis.

## 2. Progressive Definition of AKI

The pathogenesis of HRS was first delineated in 1988 by Schrier et al. [10], initially, blood accumulates in the splanchnic/portal circulation, resulting in a diminished effective arterial blood volume. In compensation, there are elevations in renin, aldosterone, catecholamines, and vasopressin, leading to systemic vasoconstriction and the retention of sodium and water in the kidneys. Subsequently, decompensated cirrhotic patients have ascites, indicative of their heightened salt retention. Ultimately, when these mechanisms fail to ensure sufficient end-organ perfusion, the characteristic “functional” kidney injury manifests, characterized by minimal or absent parenchymal kidney damage, despite elevated sCr levels and other indicators of reduced glomerular filtration rate (GFR) [11].

AKI in cirrhosis was described in 1996 by the International Club of Ascites (ICA) as a sCr rise of 50% or more from baseline to 1.5 mg/dL or higher. Oliguria and proteinuria below 500 mg/dL are further significant aspects of AKI in cirrhosis [12].

AKI has historically been characterized by a rise in sCr levels (over 1.5 mg/dL). Because it was based on a static threshold, the definition failed to take into consideration the ever-changing levels of sCr, which are crucial for differentiating between acute and chronic renal insufficiency. Despite its accessibility and low cost, the blood creatinine test has the potential to understate the extent to which cirrhotic patients’ renal function is affected due to reduced hepatic creatine synthesis, the precursor of creatinine. Less muscle mass, female gender, and less creatinine secreted by the renal tubules all contribute to lower blood creatinine levels. Hyperbilirubinemia can also significantly interfere with sCr estimation when using Jaffe’s kinetic method, leading to falsely low creatinine values. According to Nigam et al. (2016) [13], this interference is due to bilirubin reacting with the alkaline picrate reagent, which suppresses the chromogen formation needed for accurate creatinine detection. This study demonstrated that the degree of interference varies depending on the assay kit used, with some commercial kits incorporating corrective algorithms or reagents that minimize bilirubin’s impact. Thus, the reliability of creatinine measurements in icteric samples depends on the method’s ability to account for bilirubin interference.

Over the last 20 years, there have been several revisions to the criteria of AKI [5,9,14], which are illustrated in Table 1.

In 2004, the Consensus Conference of the Acute Dialysis Quality Initiative Group defined the RIFLE classification, which was based on changes in sCr or GFR and urine output. AKIN revised this categorization three years later (2007) to include all forms of acute renal damage and to add a 0.3 mg/dL absolute rise in baseline blood creatinine within 48 h as a criterion of AKI [9,17].

In 2007, there were two additional types of HRS: type 1, which is marked by a fast decline in kidney function, with a sCr level of 2.5 mg/dL or higher, or a 50% decrease in the initial 24-h creatinine clearance to 20 mL/min or lower in less than 2 weeks, often caused by an external factor; and type 2, where the progression of kidney failure did not meet the criteria for type 1. Notably, the revised diagnostic criteria did not include urine sodium or oliguria [12].

The RIFLE and AKIN criteria for AKI were further refined in 2012 by KDIGO. AKI is defined as a rise in sCr of at least 0.3 mg/dL (or 26.5 mmol/L) within 48 h, a 1.5-fold increase from baseline, which should have happened within the past 7 days, or a urine volume less than 0.5 mL/kg/h for 6 h. The most recent blood creatinine level during the last three months is used as a reference point in cases when a value from the last seven days is unavailable [5,9,18]

Patients with cirrhosis who have a urine output below 0.5 mL/kg for more than 6 h have a greater mortality rate compared to those who fulfill merely the creatinine criteria for AKI, even if reduced urine output might be a consequence of excessive salt and water retention. Diagnosis criteria developed for KDIGO include the following: increased risk of in-hospital and 30-day mortality; increased risk of multiorgan failure; and length of hospital stay [5].

The ICA-AKI criteria were developed by the ICA in 2015 when they revised the AKI classification to include cirrhosis predictors defined in Table 2. The percentage of sCr rise from baseline is the primary determinant of this categorization system. Due to the anticipated decreased urine production in cirrhotic patients as a result of excessive salt retention, the ICA also removed urine output from the updated definition of AKI [9,17].

sCr measured in the prior three months may serve as a baseline when a level acquired within the preceding seven days is unavailable. While oliguria is not part of the AKI definition in cirrhotic patients because baseline urine volume is often low in patients with cirrhosis and ascites, a study demonstrated a substantial correlation between urine output and unfavorable outcomes in individuals with HRS [12]. HRS is described by the ICA, whose revised 2015 guidelines significantly emphasize this comprehension of pathophysiology, along with clinicians’ capacity to exclude other etiologies of AKI (Table 3). The ICA guidelines additionally advocate substituting the name “HRS-1” (the acute and more severe AKI in cirrhosis that fulfills these criteria) with “HRS-AKI” (hepatorenal syndrome-acute kidney injury). Despite the near synonymy of the two words, we consistently employ HRS-AKI in this evaluation [11,21,22].

The diagnostic criteria and terminology for HRS type-1, now known as HRS-AKI, were amended by ICA in 2019. A blood creatinine level below 2.5 mg/dL does not rule out HRS-AKI diagnosis. If a functional kidney injury does not meet the criteria of HRS-AKI, it is referred to as HRS-NAKI, or non-AKI. HRS-acute kidney disease (HRS-AKD) is a subset of NAKI that occurs when the estimated glomerular filtration rate (eGFR) is below 60 mL/min/1.73 m^2^ for less than three months, and HRS-chronic kidney disease (HRS-CKD) occurs when the eGFR remains below 60 mL/min/1.73 m^2^ for more than three months [12,15]. Table 4 shows the evolution of the terms and the changes in the criteria over the years.

## 3. Diagnostic Difficulties and Renal Function Evaluation in Cirrhosis-Associated AKI

HRS-AKI is frequently induced by situations that are theoretically avoidable. Preventive strategies encompass the abstention from alcohol consumption, surveillance of sCr and electrolytes in patients receiving diuretics, administration of albumin after therapeutic paracentesis in certain conditions, provision of antibiotics during gastrointestinal bleeding episodes, prophylactic antibiotics to avert spontaneous bacterial peritonitis, albumin administration in conjunction with antimicrobial therapy in certain patients with spontaneous bacterial peritonitis, and the avoidance of nonselective beta-blockers and nephrotoxic agents, including angiotensin-converting enzyme inhibitors, angiotensin II receptor blockers, and nonsteroidal anti-inflammatory drugs [5,24].

Upon establishing the diagnosis of AKI, the etiology—whether hypovolemic AKI (prerenal azotemia), ATI, or HRS-AKI—is identified. Urinalysis is conducted to identify hematuria, proteinuria, or atypical urine sediment, hence excluding structural renal disorders [5].

The existing guidelines that characterize AKI through a relative alteration in blood creatinine levels fail to differentiate the etiologies of AKI, particularly between functional/prerenal impairment and parenchymal/tubular damage, and do not provide tissue diagnosis for validation. Kidney biomarkers such as NGAL, KIM-1, IL-18, L-FABP (liver-type fatty acid–binding protein), and CysC possess the capacity to fill several knowledge gaps about AKI, including early detection, differential diagnosis, and prognosis. Initially, many prospective investigations have shown that various kidney indicators can identify AKI within hours after damage. This facilitates the prompt detection of AKI and may influence the timing of kidney replacement therapy and the allocation of patients to more suitable levels of care. Secondly, certain biomarkers can differentiate prerenal acute kidney damage from ATI, hence restricting volume resuscitation to patients with fluid-responsive renal impairment. Third, renal biomarkers can forecast the severity of AKI by identifying individuals likely to advance to a more severe stage of AKI. The early identification of AKI, when combined with this approach, has the potential to enhance triage based on illness severity even further. Fourth, many kidney biomarkers have been linked to long-term clinical outcomes, including renal recovery rates, cardiovascular results, and overall mortality [5,11,25,26].

Urine output and sCr are the most commonly used biomarkers for assessing kidney function and continue to be fundamental assays in clinical environments for diagnosing and staging AKI. Changes in sCr levels and/or urine output serve as clinical indications of acute renal impairment. Employing a decrease in urine output as a diagnostic criterion in patients with cirrhosis and ascites presents a challenge, since these individuals often exhibit preexisting oliguric conditions with significant sodium retention while potentially preserving a reasonably normal GFR [23,27].

Conversely, patients with liver cirrhosis may exhibit elevated urine production due to diuretic therapy. Urine collection frequently lacks accuracy in clinical practice, and the utilization of kinetic alterations in sCr is pivotal for the detection of AKI in cirrhosis. The diagnosis continues to depend on the patient’s medical history and physical examination, which assesses the probability of a specific etiology of AKI prior to testing. In cirrhotic people, traditional urine markers such as albuminuria are of limited value due to hypoalbuminemia and relatively increased capillary permeability. Normal proteinuria and urine tests may not sufficiently exclude renal parenchymal problems. Kidney ultrasonography frequently excludes obstructive uropathy as a cause of AKI [17,23,28].

sCr is the most common biomarker for assessing renal function in patients with acute renal failure, regardless of the presence of cirrhosis. sCr as a renal function indicator possesses numerous limitations in clinical practice [17,28]. Patients with cirrhosis cannot have their GFR evaluated using formulas based on sCr levels. The depletion of skeletal muscle and the diminished synthesis of creatine by the liver both lead to the increased sCr levels observed in cirrhosis [12]. A significant shortcoming of creatinine-based GFR equations is that creatinine serves as a rather insensitive indicator of early acute alterations in renal function, particularly in liver patients who frequently exhibit low muscle mass. Renal injury can occur 24 to 72 h prior to an increase in blood creatinine, leading to a considerable delay in the identification of AKI. High prevalence of sarcopenia, ascites, and elevated bilirubin levels may influence sCr levels. When sCr increases, the eGFR must have decreased substantially [4,29,30]. Sole reliance on the reported estimated GFR may lead to an inadvertent underestimation of the severity of AKI or a failure to recognize AKI and CKD in patients with liver illness. A meticulous evaluation of the patient and an increased level of suspicion are essential for identifying renal disease in individuals with cirrhosis. A meta-analysis indicated that the creatinine-based computation overestimated GFR by 18 mL/min [18,31]. Consequently, sCr levels are inherently lower, resulting in an eGFR that frequently exceeds the actual GFR. There is significant interest in identifying novel indicators of renal injury to facilitate earlier detection and differentiate between renal parenchymal damage and prerenal azotemia [29].

Novel kidney biomarkers can be classified into three categories: (1) indicators of renal function, (2) markers of tubular injury, and (3) indicators of cell-cycle arrest [11,32].

### 3.1. Biomarkers for the Assessment of Kidney Function

Serum cystatin C (sCysC) has been studied to enhance the identification of diminished GFR. Researchers have investigated CysC as a potential predictor of AKI or mortality to address these limitations. The nucleated cells of the body synthesize CysC, a low-molecular-weight protein (13 kDa), which is filtered by the glomeruli and reabsorbed in the proximal tubule [4,11,33]; its elimination occurs exclusively through the kidneys’ glomerular filtration system [5,18,32,34]. It exhibits a kinetic profile analogous to creatinine, rising 12 to 24 h subsequent to the beginning of AKI [11,32]. sCysC concentrations provide a more precise assessment of GFR and are less affected by ethnicity, age, gender, sarcopenia, and diabetes compared to sCr; diminished GFR correlates with elevated CysC levels [4,5,33].

Urinary CysC has emerged as a promising biomarker for the early detection and prognosis of AKI, particularly due to its stability and independence from muscle mass. Its urinary excretion reflects tubular injury, making it potentially useful in cirrhotic patients where serum levels may be affected by liver dysfunction. Patients with cirrhosis may derive greater advantage from an eGFR calculated using CysC rather than the sCr-based method. Nonetheless, CysC may not serve as the most reliable indication of GFR in cirrhosis, as its levels are influenced by hypoalbuminemia, elevated C-reactive protein, and leukocytosis. SCr and CysC, when utilized in conjunction, yield a more precise estimation of GFR than each parameter independently. The application of CysC-based eGFR assessments in cirrhotic patients is awaiting formal permission for routine implementation [18,29,35].

Increased baseline CysC levels correlate with the onset of AKI; levels exceeding 1.3 mg/L are often linked to an elevated risk of AKI, and levels escalate with the severity of kidney disease. Analogous to NGAL, elevated CysC levels frequently preceded sCr by 48 h, and CysC has a superior correlation with measured GFR compared to sCr in individuals with DC. Levels of CysC, previous occurrences of AKI, and the degree of hyperbilirubinemia are autonomous predictors of subsequent AKI. On the other hand, CysC demonstrates a restricted capacity to distinguish between the etiologies of AKI. In hospitalized patients with cirrhosis who experience AKI, CysC may be less effective than NGAL in distinguishing the etiologies of AKI, particularly in advanced stages of HRS. CysC may serve as a superior indicator of early HRS compared to later stages characterized by tubular damage. Elevated CysC may aid in the identification of early-stage HRS; in advanced phases, the trend of CysC parallels that of sCr in forecasting outcomes.

In addition to their role in assessing renal function, some biomarkers also demonstrate prognostic value. Kim et al. demonstrated that sCysC is substantially correlated with the onset and advancement of AKI, as well as the mortality of hospitalized patients with DC. Demonstrated the usefulness of sCysC as a prognostic indicator for the onset of renal dysfunction, HRS, and acute-on-chronic liver failure (ACLF), in addition to 90-day transplant-free mortality in individuals with acutely decompensated liver cirrhosis [36]. Increased CysC levels are a reliable indicator of both short-term and long-term death. A substantial prospective cohort analysis identified CysC and Model for End-stage Liver Disease (MELD) score as the sole independent predictors of mortality after a median follow-up of 7 months [25,37]. The incorporation of sCysC into MELD components more accurately predicted death than the conventional MELD score currently utilized. The authors found that scores including sCysC might reliably predict the onset of AKI and mortality in these individuals [37].

### 3.2. Biomarkers for Defining the Phenotype of AKI (Tubular Injury Biomarkers)

Identifying the AKI phenotype is essential for formulating a therapeutic approach and enhancing clinical outcomes [32]. Novel biomarkers exclusively expressed and released during overt tubular injury can differentiate functional from structural AKI in various contexts, such as ATI vs. HRS-AKI [5,9], and show significant potential in patients with cirrhosis. The impairment of the renal tubule’s capacity to conserve sodium due to structural damage (e.g., ATI) renders urine sodium and fractional excretion of sodium (FENa) viable indicators of renal injury [14]. Likewise, additional established biomarkers (e.g., urine albumin, fractional excretion of urea) have been proposed in this context and are routinely utilized in clinical practice [22].

Regrettably, all these markers demonstrate restricted accuracy in cirrhosis. In people with HRS, urine sodium excretion typically measures less than 10 mEq/L; however, it may be elevated in those who have recently been administered diuretics. The FENa more precisely represents salt management than urine sodium alone; however, it is influenced by water reabsorption. FENa is utilized in evaluating individuals with diminished urine output; a low FENa typically signifies volume depletion, while a high FENa shows renal salt loss, as observed in ATI. FENa is a fundamental tool for nephrologists in differentiating functional from structural ATI AKI; although, it is frequently regarded as an ineffective diagnostic tool in patients with advanced liver disease. These patients exhibit nearly universal sodium retention, resulting in a FENa that is almost always below the conventional diagnostic threshold of 1%, even in individuals without AKI and occasionally in those with ATI. A FENa threshold of <1% indicates prerenal etiologies, including HRS, whereas a FENa > 1% implies structural causes of AKI, such as ATI. A recent study evaluating individuals on a liver transplant waiting list found no link between the FENa and the etiology of AKI as determined by kidney biopsy [5]. In cirrhosis, an FENa of <1% demonstrated a sensitivity of 100% but a specificity of about 14% for diagnosing prerenal causes of AKI [5,14,24,38].

Fractional excretion indices (e.g., FENa, FEUrea) offer valuable insights into renal handling of solutes and can serve as indirect indicators of both hepatic synthetic function and renal excretory capacity. In cirrhotic patients, these parameters help differentiate between pre-renal states, HRS-AKI, and intrinsic renal injury, offering a nuanced view of combined liver-kidney dysfunction. Fractional excretion of urea (FEUrea) may more effectively differentiate HRS from prerenal azotemia or ATI. FENa may be elevated due to diuretics, glycosuria, or chronic adaptation. Urea, primarily reabsorbed in the proximal renal tubule and collecting ducts, remains unaltered by diuretic administration [28,32,39]. Limited research has evaluated the efficacy of FEUrea as a substitute for FENa. Gowda et al. (2022) [40], in a prospective study involving 200 patients, examined the biomarkers FENa and FEUrea, revealing that FENa considerably surpassed FEUrea in differentiating ATI from non-ATI and HRS from non-HRS. In a limited retrospective investigation, a FEUrea of <28.16% demonstrated a sensitivity of 75% and a specificity of 83% in distinguishing HRS from non-HRS. Consequently, FEUrea may be utilized in the differential diagnosis of HRS-AKI [41].

Biomarkers indicative of renal tubular injury encompass tubular proteins released after cellular damage (N-acetyl-β-D-glucosaminidase (NAG), α-glutathione S-transferase) and tubular proteins increased by injury, including KIM-1 and NGAL. Inflammatory markers, including IL-18 and plasma proteins exhibiting diminished tubular reabsorption due to renal tubular cell injury (α1-microglobulin, β2-microglobulin, retinol binding protein), have also been utilized. AKI in cirrhosis can be assessed using urinary markers of renal tubular injury. Included are IL-18, KIM-1, NAG, L-FABP, and urine neutrophil gelatinase-associated lipocalin (uNGAL) [9,18,23,42,43,44]. All specialists concurred on the prospective function of novel urine biomarkers in the differential diagnosis of various kinds of AKI in patients with cirrhosis [5,25,45].

NGAL is a 25 kDa glycoprotein synthesized by neutrophils and epithelial cells, including renal tubular cells, rendering it an optimal marker for tubular injury, with levels increasing rapidly in the early stages of tubular damage. NGAL is produced by several organs and is filtered by the glomerulus for reabsorption by the proximal tubule [21,29]. In the event of renal damage, NGAL is synthesized in the loop of Henle, distal tubule, and collecting ducts, and is thereafter excreted in the urine. NGAL accumulates in the tubule and is detectable as early as one hour post-injury, with accumulation correlating to the injury’s severity [46].

A significant benefit of NGAL is its ability to rise prior to an increase in sCr by 1–3 days in instances of AKI, and it is more readily quantifiable than alterations in urine output. NGAL is released during acute tubular damage and is not invariably high in CKD. This aids in distinguishing the etiology of AKI superimposed on an unidentified baseline or CKD. Numerous studies indicate that increased NGAL levels predict mortality, irrespective of the MELD score. Since NGAL is also synthesized by neutrophils, it may be raised during infections, particularly urosepsis, potentially leading to false-positive results [21,29,47].

Numerous investigations have shown that uNGAL possesses great precision in distinguishing ATI from HRS–AKI and hypovolemic AKI. It can be utilized for the differential diagnosis of AKI phenotypes. It might distinguish among subgroups of renal injuries, such as prerenal AKI, HRS, and ATI [22]. There is evidence indicating that they can significantly differentiate intrinsic renal damage from hemodynamic changes resulting from fluid loss. uNGAL is acknowledged as a robust biomarker for the detection of AKI, the distinction of its cause, and as a prognostic indicator in diverse clinical contexts. uNGAL exhibits optimal performance following two days of plasma expansion with albumin, as advised in the treatment of AKI [29]. Nonetheless, NGAL levels also elevate in sepsis, the severity of liver illness, and urinary tract infections, whereas measuring uNGAL is challenging in cases of anuria [25].

The uNGAL threshold over 220 µg/g of creatinine [9] (but surpassing 244 µg/g of creatinine) [4] exhibited the highest diagnostic precision for ATI. Research indicates that uNGAL serves as an independent predictor of short-term mortality. Recent studies indicate that uNGAL measured on day 3 is the most precise method for distinguishing between ATI and alternative causes of AKI. This study encompassed 320 patients admitted for DC and determined that this measurement exhibited the best level of accuracy [18]. In a prospective cohort study by Fagundes et al. [48], it was found that among 241 cirrhosis patients, uNGAL levels are significantly elevated in ATI patients compared to those with prerenal AKI, CKD, and HRS.

In a recent study conducted by Hamdy et al. [49], uNGAL > 143 (ng/mL) can differentiate intrinsic AKI from HRS in cirrhotic patients, with 75% sensitivity and 80% specificity. uNGAL is a promising biomarker for AKI, demonstrating improved performance compared to urine output and sCr. The diagnosis time for AKI is decreased, with a 15-fold increase in uNGAL levels observed within 2–4 h post-insult compared to non-AKI cases. In cases of serious injury, elevation may persist for as long as 5 days following the initial trauma. Slack et al. [50] found that in liver cirrhosis, uNGAL levels in AKI patients were significantly elevated compared to those in non-AKI patients. Elevated uNGAL may correlate with irreversible AKI, as levels are greater in persistent AKI compared to transitory AKI; furthermore, increased uNGAL is linked to the progression of AKI. Serum NGAL levels may predict AKI following paracentesis, and NGAL levels in ascitic fluid may identify patients with or at risk for spontaneous bacterial peritonitis.

uNGAL levels are minimal for prerenal etiology, moderate for HRS, and markedly elevated for ATI, despite some overlap among the etiologies. The distinctions may indicate a continuum from the absence of tubular injury (prerenal), to exacerbated kidney vasoconstriction (HRS), culminating in substantial ischemia injury (ATI) [25,51].

Gambino et al. (2023) [20] conducted a study on the diagnostic and prognostic efficacy of uNGAL in patients with cirrhosis and AKI. This study involved 162 patients, demonstrating that uNGAL is a dependable biomarker for the differential diagnosis of AKI, predicting the response to treatment for HRS-AKI, and predicting in-hospital mortality. uNGAL levels were markedly elevated in ATI compared to other forms of AKI. This study indicated that uNGAL is a superior urine biomarker for differentiating between ATI-AKI and HRS-AKI, and it can significantly enhance the diagnosis of AKI in patients with cirrhosis. The optimal threshold of uNGAL for distinguishing ATI AKI from other types of AKI was determined to be 220 ng/mL, with a sensitivity of 89% and a specificity of 78%. The other finding of this study is that uNGAL levels predict treatment response to terlipressin and albumin in patients with HRS-AKI, with elevated uNGAL levels correlating with markedly reduced response rates [20].

Studies connected to NGAL have demonstrated its potential as a predictive biomarker for predicting the likelihood of AKI development, severity, the necessity for RRT, intensive care unit admission, hospital mortality, and progression to CKD. Belcher et al. [52] demonstrated the efficacy of uNGAL and its association with the course of AKI and death. A meta-analysis conducted by Puthumana et al. [53] confirmed that uNGAL can effectively predict short-term mortality in patients with liver cirrhosis.

The most conclusive investigation on uNGAL in cirrhosis and AKI was conducted by Huelin et al. [54]. The authors examined 320 consecutive cases of AKI in cirrhosis utilizing a standardized procedure and assessed uNGAL on days 1, 3, 7, and 14 of hospitalization. The day 3 uNGAL level (post-volume challenge) effectively differentiated ATI from other forms of AKI, predicted the progression of AKI stages, and anticipated 28-day mortality. In the context of a diagnostic methodology, Huelin et al. proposed that NGAL may be beneficial for the differential diagnosis of AKI and for predicting outcomes in cirrhosis [11].

NAG is derived from the lysosomes of proximal tubules; thus, elevated urine NAG levels indicate proximal tubule damage and compromised lysosomal integrity. In contrast to NGAL, NAG possesses a substantial molecular weight (140 kDa) and is not subject to glomerular filtration; hence, elevated urinary concentrations are improbable to arise from extrarenal origins. Numerous investigations have demonstrated that urine NAG serves as a sensitive indicator of tubular damage [32]. A prospective cohort study by Yoo et al. (2021) [55] evaluated the role of urinary NAG, a marker of tubular injury, in cirrhotic patients with AKI. Conducted across 11 tertiary hospitals in Korea and involving 262 patients, this study found that urinary NAG levels increased significantly with AKI severity and were notably higher in patients who failed to recover from AKI or who experienced death or liver transplantation within three months. Elevated NAG was identified as an independent predictor of short-term, transplant-free survival, particularly in patients with lower MELD scores or Child–Pugh A/B cirrhosis. However, NAG was not effective in distinguishing between different AKI phenotypes (such as prerenal AKI, ATI, or HRS) and did not predict response to terlipressin in patients with HRS-AKI. These findings support the prognostic value of urinary NAG in cirrhotic AKI, while highlighting its limitations in diagnostic stratification and therapeutic guidance [55].

KIM-1 is a transmembrane glycoprotein that detaches from the tubular epithelial surface of the proximal tubules within hours following kidney damage [11,29,32]. A new meta-analysis indicates that urinary KIM-1 measurement is a sensitive and specific marker of kidney injury, albeit a weak predictor of nonischemic AKI. KIM-1 levels start to elevate 24 h post-ischemia damage and reach their zenith at 48 h. The relative delay in the elevation of KIM-1 may restrict early intervention. KIM-1 is additionally secreted during the healing of the proximal tubule, confounding the explanation of increased levels [29,56].

IL-18 is a pro-inflammatory cytokine produced in the proximal tubule and released upon tubular damage [9,32]. The concentration of this substance in urine rises following acute ischemia damage to the proximal tubule, a trend that appears unaffected by urinary infections, sepsis, or CKD. Patients with cirrhosis and ATI exhibit markedly elevated IL-18 levels in comparison to other etiologies of renal damage. Similar to NGAL, evidence indicates a relationship between IL-18 levels and short-term mortality [9,11,32].

L-FABP is a free fatty acid transporter located in the proximal tubule, released into urine after sepsis and AKI, and may be influenced by infection and liver disease. Human studies indicate that urine L-FABP may serve as a predictor for AKI or sepsis worsened by AKI, with its levels potentially influenced by infection or hepatic illness. Moreover, urine L-FABP levels are elevated in individuals with CKD, indicating its function as an antioxidant and renoprotective agent [11,32]. A study by Juanola et al. (2022) [57] investigated the prognostic value of urinary L-FABP in patients with DC, focusing on its association with ACLF and mortality. The prospective cohort included 444 hospitalized patients with DC, divided into a study cohort (305 patients) and a validation cohort (139 patients). Urinary L-FABP levels were measured upon admission, alongside plasma L-FABP and uNGAL. This study found that urinary L-FABP, but not plasma L-FABP, correlated with 3-month survival on univariate analysis. Multivariate analysis revealed that urinary L-FABP and the Model for End-Stage Liver Disease with sodium (MELD-Na) score were the only independent predictors of prognosis. Urinary L-FABP levels were higher in patients who developed ACLF and in those with AKI, particularly ATI. These findings were consistent across both cohorts. In contrast, urinary NGAL predicted outcomes only on univariate analysis. This study concluded that urinary L-FABP is a promising prognostic biomarker for 3-month mortality and ACLF development in patients with DC.

Insulin-like growth factor binding protein 7 (IGFBP7) and tissue inhibitor of metalloproteinases-2 (TIMP-2), a dual-component urine biomarker indicative of cell-cycle arrest, constitute the final grouping of renal biomarkers. An impartial proteomic screen (a comprehensive, unbiased analysis of a wide range of proteins in biological samples without pre-selecting specific targets) in human populations identified it as effective in detecting AKI and predicting clinical outcomes [11].

[TIMP 2] × [IGFBP7] is a newly identified cell-cycle arrest protein produced in renal tubular cells under conditions of cellular stress or damage. TIMP-2 suppresses matrix metalloproteinase activity, influencing cell cycle control, and binds to α3β1 integrin on endothelial cell surfaces, hence suppressing endothelial cell proliferation and angiogenesis. IGFBP7 is a secreted protein that modulates the bioavailability of IGFs via direct binding. While [TIMP 2] × [IGFBP7] is FDA authorized as NephroCheck^®^, for risk assessment of moderate to severe AKI in adult ICUs, it appears to be just one study that addresses the relevance of this marker in cirrhosis, which is not elaborated upon further [58,59].

In a study by Suksamai et al. (2025) [60], urinary TIMP-2, particularly when combined with IGFBP7, was evaluated as a sensitive biomarker for detecting early kidney injury following moderate-volume paracentesis in patients with ascites due to DC. The randomized trial compared 3 L and 5 L paracentesis groups and found that patients in the 5 L group had significantly higher post-procedure urinary levels of TIMP-2·IGFBP7, with 48% showing values greater than 2. Additionally, increases in TIMP-2 alone were observed in 32%, and the TIMP-2/urine creatinine ratio rose in 76% of cases. These changes occurred without corresponding drops in GFR, indicating subclinical tubular stress. TIMP-2·IGFBP7 levels greater than two were associated with hemodynamic instability, reinforcing the role of these markers in identifying early renal stress. The findings support the use of urinary TIMP-2·IGFBP7 as a predictive tool for AKI risk in cirrhotic patients undergoing paracentesis and suggest that even moderate fluid removal volumes, like 5 L, can trigger renal stress before traditional kidney function markers detect injury [60].

Although many biomarkers such as KIM-1, IL-18, and L-FABP remain largely confined to research settings, NGAL stands out as one of the few with commercially available point-of-care diagnostic platforms. Both plasma and urinary NGAL assays have been integrated into clinical protocols in certain settings, supporting its utility beyond research.

**Figure 1:**
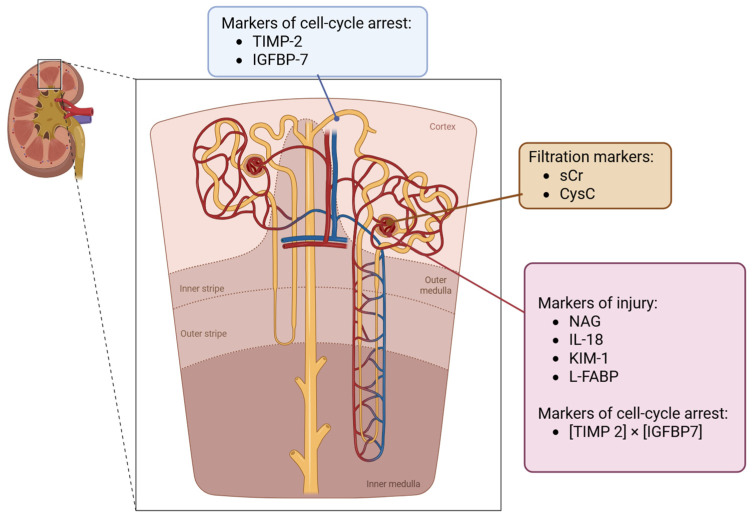
Biomarkers for defining the phenotype and function of AKI.

In a study involving 112 patients with cirrhosis with either prerenal AKI, HRS, or ATI, Belcher et al. evaluated uNGAL, IL-18, KIM-1, and L-FABP, concluding that each biomarker effectively distinguished ATI from non-tubular causes of renal damage, with NGAL exhibiting superior performance. The probability of developing ATI escalated with a greater number of positive biomarkers, as exemplified in Figure 1 [52].

One of the most intriguing aspects of this field is the capacity of biomarkers to predict treatment response. Identifying patients likely to respond to medication might enhance patient selection, hence mitigating potentially severe side effects in non-responders. A recent, prospective, observational study indicated that reduced uNGAL levels predicted the response to terlipressin in HRS-AKI. This conclusion was not evidenced in a prior cohort analysis; hence, additional confirmation is necessary [4,27].

Ultimately, biomarkers may play a crucial role in precisely predicting the prognosis of patients with cirrhosis and AKI, which could have significant consequences for transplant eligibility and the assessment of treatment futility. Conventional scoring systems like MELD fail to reliably forecast prognosis in cases of AKI and ACLF. In populations with a high prevalence of AKI patients, uNGAL correlates with 28-day and 90-day mortality, independent of the MELD score, and may enhance existing predictive metrics such as MELD or CLIF Consortium ACLF score (CLIF-C ACLF) [4].

While urinary biomarkers may offer advantages in detecting tubular injury, caution is warranted when interpreting their superiority over serum markers in liver cirrhosis. The impaired hepatic synthesis of several serum biomarkers can confound their interpretation, making urinary markers more attractive in theory, but further validation is required before a definitive conclusion can be drawn.

## 4. Conclusions

AKI adversely affects survival; thus, it is imperative to implement strategies to prevent circumstances that result in renal damage. The correlation between AKI and nephrotoxic agents, extensive paracentesis, and infections is acknowledged and has guided preventive strategies and early detection among specialist clinicians. Nevertheless, there is a scarcity of research that has thoroughly examined how AKI in patients with cirrhosis can be mitigated through these interventions.

Contemporary methods for diagnosing and monitoring the progression of AKI predominantly depend on sCr levels, which are not an optimal indicator of GFR. Distinguishing between HRS-AKI and ATI is sometimes challenging and may necessitate a kidney biopsy. The approach to kidney biopsy is fraught with challenges due to associated risks, costs, and a scarcity of pathological expertise. Biomarkers like NGAL may distinguish between HRS-AKI and ATI, although the routine use of NGAL in clinical settings is not yet widely implemented in standard clinical practice. Consequently, there is a necessity for readily accessible biomarkers to diagnose and monitor the progression of AKI, distinguish between HRS-AKI and ATI, and ascertain the reversibility of kidney failure.

Beyond early diagnosis, several biomarkers such as NGAL, TIMP-2/IGFBP7, and CysC have shown potential to guide therapeutic decisions by identifying patients at higher risk of progression, thereby informing the timing of interventions such as fluid management, avoidance of nephrotoxins, and early initiation of renal support.

Liver transplantation is the final treatment for HRS-associated AKI; nevertheless, it remains uncertain which patients benefit from liver transplantation alone and in which cases combination liver-kidney transplantation should be performed. Emerging pharmacological therapies, such as terlipressin, have shown promise in reversing HRS-AKI in selected patients. Furthermore, the existing MELD-based allocation system for liver transplantation diminishes the priority for patients with HRS-AKI who exhibit a positive response to pharmacological intervention, indicating the necessity to uphold a high priority for liver transplantation in patients with HRS-AKI, regardless of their response to treatment resulting in decreased sCr levels. No research has been performed utilizing patient-reported outcomes (with a focus on subjective experiences, like symptoms or emotional well-being; they complement clinical data by offering insights into how patients experience their illness and treatment), an objective that is increasingly mandated by regulatory authorities. Consequently, the development of patient-reported outcomes for individuals with cirrhosis and AKI is a significant future endeavor. Additionally, well-designed randomized controlled trials are needed to validate the clinical utility of biomarker-guided approaches for diagnosing, stratifying, and managing AKI in this complex patient population.

## Figures and Tables

**Table 1:** Evolution of AKI criteria.

Criteria	Stage	sCr or GFR Criteria	Urine Output Criteria	References
Risk, Injury, Failure, Loss and End-stage Kidney Disease (RIFLE) criteria in 2004	Stage 1 (Risk)	Increased sCr ≥ 1.5 × baseline or GFR decreased >25%	<0.5 mL/kg/h for ≥6 h	[9,15,16]
	Stage 2 (Injury)	Increased sCr ≥ 2 × baseline or GFR decreased >50%	<0.5 mL/kg/h for ≥12 h	
	Stage 3 (Failure)	Increased sCr ≥ 3 × baseline or GFR decreased >75%	<0.3 mL/kg/h for ≥24 h	
	Loss	Persistent acute renal failure > 4 weeks	-	
	End-stage	Complete loss of kidney function > 3 months	-	
Acute Kidney Injury Network (AKIN) in 2007	Stage 1	Increased sCr ≥ 1.5 × baseline or ≥0.3 mg/dL within 48 h	<0.5 mL/kg/h for ≥6 h	
	Stage 2	Increased sCr ≥ 2 × baseline	<0.5 mL/kg/h for ≥12 h	
	Stage 3	Increased sCr ≥ 3 × baseline	<0.3 mL/kg/h for ≥24 h or anuria ≥ 12 h	
Kidney Disease: Improving Global Outcomes (KDIGO) in 2012	Stage 1	Increased sCr ≥ 1.5–2 × baseline or ≥ 0.3 mg/dL	<0.5 mL/kg/h for ≥6–12 h	
	Stage 2	Increased sCr ≥ 2–3 × baseline	<0.5 mL/kg/h for ≥12 h	
	Stage 3	Increased sCr ≥ 3 × baseline or sCr ≥ 4.0 mg/dL	<0.3 mL/kg/h for 24 h or anuria ≥ 12 h	

**Table 2:** ICA-AKI criteria and AKI staging.

Category	Criteria	References
Definition of baseline sCr	A value of sCr obtained in the previous 3 months. In patients with more than one value, the closest and lowest to hospital admission should be used. In patients without a previous value, the admission value should be used as baseline.	[4,9,19,20]
Definition of AKI	Increase in sCr ≥ 0.3 mg/dL (26.5 µmol/L) within 48 h OR Increase in sCr ≥ 50% from baseline within the prior 7 days.	
AKI Staging		
Stage 1A	Increase in sCr ≥ 0.3 mg/dL (26.5 µmol/L) to a value < 1.5 mg/dL (133 µmol/L) from baseline at AKI diagnosis.	
Stage 1B	Increase in sCr ≥ 0.3 mg/dL (26.5 µmol/L) to a value ≥ 1.5 mg/dL (133 µmol/L) from baseline at AKI diagnosis.	
Stage 2	Increase in sCr 2-fold to 3-fold from baseline.	
Stage 3	Increase in sCr ≥ 3-fold from baseline OR sCr ≥ 4.0 mg/dL (353.6 µmol/L) with an acute increase of ≥0.3 mg/dL (26.5 µmol/L) OR Initiation of renal replacement therapy (RRT).	

**Table 3:** 2015 ICA diagnostic criteria for HRS.

Confirmed presence of cirrhosis and ascites
AKI according to ICA-AKI criteria, defined as either an increase in sCr of ≥0.3 mg/dL within 48 h, or a ≥50% increase from baseline presumed to have occurred within 7 days
No improvement in kidney function after 48 h of stopping diuretics and administering intravenous albumin at a dose of 1 g/kg (up to 100 g)
No evidence of shock
No recent use of nephrotoxic medications
No obvious signs of structural kidney damage (e.g., proteinuria < 500 mg/day, fewer than 50 red blood cells per high-power field in urine, and normal kidney ultrasound findings)

Note: The guidelines emphasize the importance of further research into urinary biomarkers to help distinguish HRS from ATI.

**Table 4:** Evolution of definitions and criteria for AKI.

Syndrome/Criteria	Old Term	Definition	HRS Prerequisites	New Term	References
AKI	HRS-1	- Stage 1: Increase in baseline sCr of either ≥0.3 mg/dL in 48 h or ≥1.5–1.9 × baseline in the last 7 d or urinary output ≤ 0.5 mg/kg body weight in ≥6 h - Stage 2: ≥2–2.9 × baseline sCr - Stage 3: ≥3 × baseline sCr or sCr ≥ 4 mg/dL or KRT	- Decompensated cirrhosis (DC) - Absence of shock - No treatment with nephrotoxic medications - No response to volume expansion - Absence of parenchymal disease (proteinuria: >500 mg/d; hematuria: <50 RBCs per HPF) - Suggestion of kidney vasoconstriction with FENa < 0.2%	HRS-AKI	[12,23]
AKD	HRS-2	eGFR < 60 mL/min per 1.73 m^2^ for <3 mo	Same as AKI	HRS-AKD	
CKD	N/A	eGFR < 60 mL/min per 1.73 m^2^ for ≥3 mo	Same as AKI	HRS-CKD	
Serum Creatinine Criteria Evolution	N/A	- 1996: sCr > 1.5 mg/dL - 2007: sCr ≥ 2.5 mg/dL - 2015: Increase in sCr ≥ 0.3 mg/dL in 48 h or ≥50% from baseline - 2019: Baseline sCr < 3 months	Varies by year, with evolving inclusion of structural kidney disease, urine sodium levels, and hemodynamic parameters	N/A	
Urine Sodium	N/A	- 1996: <10 mEq/L - 2007: <30 mEq/L - 2015: FENa < 0.2%	Absence of structural kidney disease	N/A	
Urine Volume	N/A	- 1996: <500 mL/day - 2015: <0.5 mL/kg/h for 6 h	Absence of structural kidney disease	N/A	
Urine Sediment	N/A	- 1996: No proteinuria - 2015: No significant proteinuria	No active urinary sediment	N/A	

N/A—not applicable.

## Abbreviations

The following abbreviations are used in this manuscript:

ACLF	Acute-on-chronic liver failure
AKI	Acute kidney injury
AKIN	Acute Kidney Injury Network
ATI	Acute tubular injury
CKD	Chronic kidney disease
CLIF-C ACLF	CLIF Consortium ACLF score
CysC	Cystatin C
DC	Decompensated cirrhosis
eGFR	Estimated glomerular filtration rate
FENa	Fractional excretion of sodium
FEUrea	Fractional excretion of urea
GFR	Glomerular filtration rate
HCC	Hepatocellular carcinoma
HRS	Hepatorenal syndrome
HRS-AKD	HRS-acute kidney disease
HRS-CKD	HRS-chronic kidney disease
HRS-AKI	HRS-acute kidney injury
HRS-NAKI	Hepatorenal syndrome–non-acute kidney injury
ICA	International Club of Ascites
IGFBP7	Insulin-like growth factor binding protein 7
IL-18	Interleukin-18
KDIGO	Kidney Disease: Improving Global Outcomes
KIM-1	Kidney injury molecule-1
L-FABP	Liver-type fatty acid-binding protein
MELD	Model for End-stage Liver Disease
MELD-Na	MELD with sodium
NAG	N-acetyl-β-D-glucosaminidase
NGAL	Neutrophil gelatinase-associated lipocalin
RIFLE	Risk, Injury, Failure, Loss and End-stage Kidney Disease
RRT	Renal replacement therapy
sCr	Serum creatinine
sCysC	Serum cystatin C
TIMP-2	Tissue inhibitor of metalloproteinases-2
uNGAL	Urine neutrophil gelatinase-associated lipocalin
